# Mapping the Evolutions and Trends of Literature on Wayfinding in Indoor Environments

**DOI:** 10.3390/ejihpe11020042

**Published:** 2021-06-18

**Authors:** Hessam Ghamari, Ayyoob Sharifi

**Affiliations:** 1 Interior Design Program, Department of Family and Consumer Sciences, College of Health and Human Development, California State University Northridge, Northridge, CA 91330, USA; 2 Network for Education and Research on Peace and Sustainability (NERPS), Graduate School of Humanities and Social Sciences, Hiroshima University, Hiroshima 739-8511, Japan; Sharifi@hiroshima-u.ac.jp

**Keywords:** wayfinding, indoor environments, navigation, bibliometric analysis, spatial orientations

## Abstract

Research on indoor wayfinding has increased in number and significance since the 1980s. Yet, the information on wayfinding literature is now difficult to manage given its vast scope and spread across journals, institutions, disciplines, and themes. While there is an increasing number of publications within this rapidly growing field of research, there are limited review studies in the field, and there is still missing an overall analysis of the current state of wayfinding literature and its evolution. The main objective of this study is to present a bibliometric analysis of about forty years of research on indoor wayfinding to provide an overview of the research landscape. The final database of the study contained 407 publications. VOSviewer was used as a science mapping software tool to identify major focus areas and to identify influential authors, publications, and journals using various network analysis techniques, such as term co-occurrence, co-citation, and bibliographic coupling. Similar co-occurrence analysis was used to understand how the intellectual base of the field has evolved over time and what the major themes are that have contributed to this evolution. The results show that this field has initially been mainly focused on few themes but has later become more diversified to acknowledge the multi-dimensional characteristics of indoor wayfinding. While spatial knowledge acquisition and cognitive maps are still dominant core areas, there are topics, such as signage, isovists, and the use of eye-tracking and virtual reality, that still need to be further investigated.

## 1. Introduction

Today, there is a large body of literature available on wayfinding. For instance, a simple search on Google Scholar by using the term “wayfinding” shows thousands of scholarly articles (55,800). Similar Google search for terms, including “wayfinding theory”, “wayfinding principles”, and “wayfinding guidelines” show 28,000, 19,600, and 16,700 results, respectively. These numerous search results of multiple aspects of wayfinding highlight its importance as it continues to capture the interest and attention of people from multiple disciplines (e.g., architects, designers, planners, academic researchers, neuroscientists, psychologists, gerontologists, and graphic designers). Wayfinding has been defined by researchers from different disciplines in various ways. Generally, wayfinding is associated with the process of making a decision on a route and locomotion from one point to a destination in a physical environment when the destination is not directly visible (e.g., Chen et al. [1], Kuliga et al. [2]). Downs and Stea [3] define wayfinding as the ability to orient, monitor, choose a route, and recognize a goal while navigating an environment. Arthur and Passini [4] defined wayfinding as an outcome of both cognitive functions (such as problem-solving and decision making) and behaviors (such as navigation and decision execution). Golledge [5] believes a person finds a way through different processes, such as estimating turn angles, segment lengths, and directions of movement, identifying en route and distant landmarks, and embedding the route to be taken in some larger reference frame. Cubukcu [6] defines wayfinding as “the spatial knowledge about one’s current location and destination, and the spatial relation between them”. Karimi [7] also refers to wayfinding as the act of traveling while on a chosen route.

Indoor wayfinding can cause challenges for people, particularly in complex environments like hospitals, airports, and office buildings [4]. The literature shows that wayfinding is an issue in both outdoor and indoor environments. Indoor wayfinding in public complex environments, such as large hospitals and airports, has a potential negative impact on users’ physiological and psychological health and well-being. The literature shows that wayfinding problems and navigation errors in unfamiliar environments cause frustration, anxiety, irritation, and stress [8,9]. Eagle [10] indicated that wayfinding aids in interior design environments can improve the sense of well-being. Additionally, appropriate wayfinding can contribute to stress reduction, functional efficiency, better accessibility for visitors, safety, patient independence, and cognitive skill improvement in spatial understanding [4]. Wayfinding issues cause major difficulties for the aging population due to visual impairments, cognitive decline, and limited physical mobilities (e.g., [11,12,13,14,15,16,17,18,19]).

One study reviewed and synthesized the published literature on wayfinding in interior environments and identified two broad factors of user factors and environmental factors [20]. This study categorized user factors into three domains: (1) wayfinding cognition, (2) wayfinding behavior, and (3) individual and group differences. It also classified environmental factors into two domains: (1) environmental elements and (2) environmental cues.

### 1.1. User Factors

This section reviews the literature on indoor wayfinding as it relates to user factors.

#### 1.1.1. Wayfinding Cognition

Cognitive functions refer to mental processes that involve the acquisition of knowledge, the manipulation of information, and reasoning. Memory, perception, decision making, language abilities, learning, and attention are domains of cognitive function. Multiple studies regarding wayfinding cognition have relied on Siegel and White’s [21] classifications of spatial memories, which include survey knowledge, landmark knowledge, and route knowledge. Survey or configurational knowledge is “knowledge of the overall spatial layout of the environment” and the spatial relationships between places and objects. Landmark knowledge consists of the knowledge of places and objects according to their appearances in the environment “without knowledge of their relative spatial relationship [22,23]. Siegel and White [21] defined route knowledge as the sequence knowledge connecting places or objects.

The cognitive map is a concept that relates to spatial memories. Weisberg and Newcombe [24] define cognitive maps as “spatial representations that contain qualitative metric information about large-scale environments, and which can be used to generate novel shortcuts or to take detours” (p. 768). The cognitive map concept was first introduced by Edward Tolman [25]. He found that rats can create a mental map that assists them in finding their destinations by taking alternative routes. This was named the “cognitive map”. Other studies [24,26,27] argue that a cognitive map is a spatial representation of the environment that includes Euclidean information, spatial layout, and affordances of the environment. Golledge et al. [28] defines cognitive mapping as “the process of extracting information from an external environment and storing it in the mind” [28] (p. 126). Some other studies proposed that wayfinding relates to information processing that involves attention, memorizing, learning, reasoning, problem-solving, and decision-making [29,30,31,32,33].

#### 1.1.2. Wayfinding Behavior

Jamshidi et al. [20] classified two aspects of wayfinding behavior as behavioral performance and navigation pattern. Behavioral performance is in regard to navigating performance in the environment with the measurement of distance, speed, and time. Navigation pattern refers to a person’s preference in selecting a certain physical route, zone, or space while navigating an environment. Hund [34] found that visuospatial memory plays an important role in wayfinding. There have been some studies showing that males were faster in completing wayfinding tasks [35,36,37]. Kober and Neuper [38] found underlying cognitive process differences between males and females during wayfinding.

#### 1.1.3. Individual and Group Differences

Multiple studies have investigated the individual and group differences during wayfinding in indoor environments. Some studies found that females rely more on landmarks than males during indoor wayfinding (e.g., [36,37]). Several other studies found that females prefer egocentric reference frames and males prefer allocentric reference frames [1,39,40]. Additionally, the literature suggests that aging is associated with cognitive function declines and results in more wayfinding errors. Some studies showed that older adults acquire less spatial knowledge during wayfinding [41,42,43]. Studies also suggest that elderlies perform more slowly in spatial tasks [43,44].

### 1.2. Environmental Factors

This section reviews the identified environmental elements and cues of indoor wayfinding as it relates to environmental factors. Kevin Lynch [45] studied peoples’ behavior in urban settings and concluded that some environmental factors (path, node, district, edge, and landmark) form the cognitive maps. These five environmental factors have been adapted and translated to interior environments (e.g., [14,22,33,46,47,48]). In addition to these environmental factors, signs, maps, and floor plan configurations have been identified as the other environmental factors that contribute to wayfinding [9,46,49,50,51,52,53,54,55,56]. The review of the literature shows that research on indoor wayfinding has increased in number and significance since the 1980s. However, the information on wayfinding literature is now challenging to manage, given its vast scope and spread across journals, institutions, disciplines, and themes. It should be noted that while in the past decade, there has been an overall increase in the number of academic publications in almost all research fields, the topic of indoor wayfinding has received specific attention, due to several potential reasons. First, there is an increasing number of publications that show that wayfinding problems and navigation errors in unfamiliar complex environments cause frustration, anxiety, irritation, and stress, particularly in a growing number of older populations. Cognitive impairment is becoming a common issue for the increasing numbers of the elderly population and wayfinding issues can negatively impact health outcomes. Therefore, there is a crucial need for increased research on factors and attributes that support late-life health and well-being, and which guarantee more independence for these vulnerable groups. Second, technological advances and using virtual/augmented reality, eye-tracking, and artificial intelligence are much more prevalent in investigating the complexity of indoor wayfinding in real and virtual environments. Third, there is a remarkable rise in the prevalence of complex indoor environments, such as hospitals, airports, and shopping malls around the world, which highlights the necessity of better-designed strategies and decision-making.

Wayfinding is a dynamic, multifaceted concept and it relates to many other disciplines; this makes it a challenge to conduct a comprehensive review of the topic and its evolution. It is essential to know what the major themes are that contributed to the evolution. While there is an increasing number of publications within this rapidly growing field of research, there have been limited efforts to mapping the evolution and current trends in indoor wayfinding. A recent study provided a narrative review of wayfinding in interior environments [57]. Another study reviewed and synthesized the published literature on wayfinding in interior environments and identified two broad factors of user factors and environmental factors [20]. While these two studies provide a better understanding of the evolution of theories of wayfinding and synthesize the domains of indoor wayfinding, there is no study in place that provides an overall review of the published literature and the conceptual evolution of wayfinding literature over time.

The main objective of this study was to present a bibliometric analysis of about forty years of research on indoor wayfinding. This analysis can provide thematic analysis and science mapping, as well as identifying the most influential authors, universities, journals, and countries that have conducted research in the field. Interested readers are referred to [58] for more details regarding bibliometric analysis. The results of such analyses can be used for different purposes. For instance, they could be used to identify understudied topics and themes that need further research. In addition, researchers new to the field can refer to the outputs as a one-stop source to obtain a relatively quick understanding of the field and its structure. This would help them in their research design process. It must be noted that bibliometric analysis is different from systematic reviews and it aims to show the overall landscape of the research and highlight major topics that have been dealt with generally and in different periods. Some of the sub-topics highlighted in this study warrant a more detailed analysis that can only be achieved through a systematic review.

This paper is divided into four sections. After the introduction, the materials and methods are discussed in Section 2. In Section 3, the results of the bibliometric analysis are reported and interpreted. Section 4 presents the conclusions of the study with a discussion on the main emerging topics in the literature, limitations of the study, and recommendations for future studies.

## 2. Materials and Methods

The major phases of the study include creating the database and the Visualizing Scientific Landscape (VOSviewer) analysis. These phases are discussed here.

### 2.1. Creating Database

The first phase was to create a database that included the relevant studies of interior wayfinding. For this purpose, a wide-range literature search string was used that consisted of keywords relevant to wayfinding in interior environments. The search terms were selected based on the authors’ knowledge of the field, and also through the analysis of existing review papers on the topic. When designing the search string, it was also considered that different variants of terms, such as “wayfinding”, may have been used in the literature.

The following is the search string used in this study: TS = ((“wayfinding” OR “way*finding” OR “indoor wayfinding” OR “interior wayfinding” OR “indoor way finding” OR “interior way finding” OR “indoor navigation” OR “interior navigation” OR” spatial*cognition” OR “spatial*behavior” OR “route*planning” OR “Cognitive*map*”) NOT (“pedestrian*” OR “urban*” OR “outdoor*”)).

The search results were limited to articles in addition to limiting to the “topic section” (title, keywords, and abstracts) to identify publications mainly focusing on indoor wayfinding. While this search might not retrieve all related publications, it found the publications in which the researcher(s) have the main focus of wayfinding and navigation in a physical indoor environment. Since the focus of this study was indoor environments, the studies that focused on the outdoor urban environment were filtered out by adding exclusion terms (“pedestrian”, “urban”, and “outdoor”) to the query. The search was conducted on 19 April 2021, based on the abstracts, keywords, and titles of research papers (only in English) that have been indexed since 1965. There were 1416 papers retrieved from the following Web of Science (WoS) databases: Science Citation Index Expanded, Social Sciences Citation Index, Arts and Humanities Citation Index, and Emerging Sources Citation Index. These databases have been selected for two main reasons: (1) they are well-recognized in academia for archiving quality peer-reviewed research; (2) the software tools available for text mining and bibliometric analysis can only process bibliographic outputs generated by these databases.

The initial search findings returned some publications related to robotics and computer science information systems, as terminology is shared between these research fields. Therefore, irrelevant publications that lacked a relationship with wayfinding were filtered out from the database. Overall, 1044 publications were removed from the database after careful review of the abstracts, keywords, and titles by the authors. Using the same keywords of the search string, a hand search was also conducted with different journals related to the field, including the Journal of Environment and Behavior, Health Environment Research and Design Journal, the Journal of Environmental Psychology, Environment and Behavior, the Journal of Experimental Psychology: Learning, Memory, and Cognition, the Cognitive Psychology Journal, and Environment and Planning B: Urban Analytics and City Science. An additional 35 articles obtained via the hand search were added to the database.

**Figure 1:**
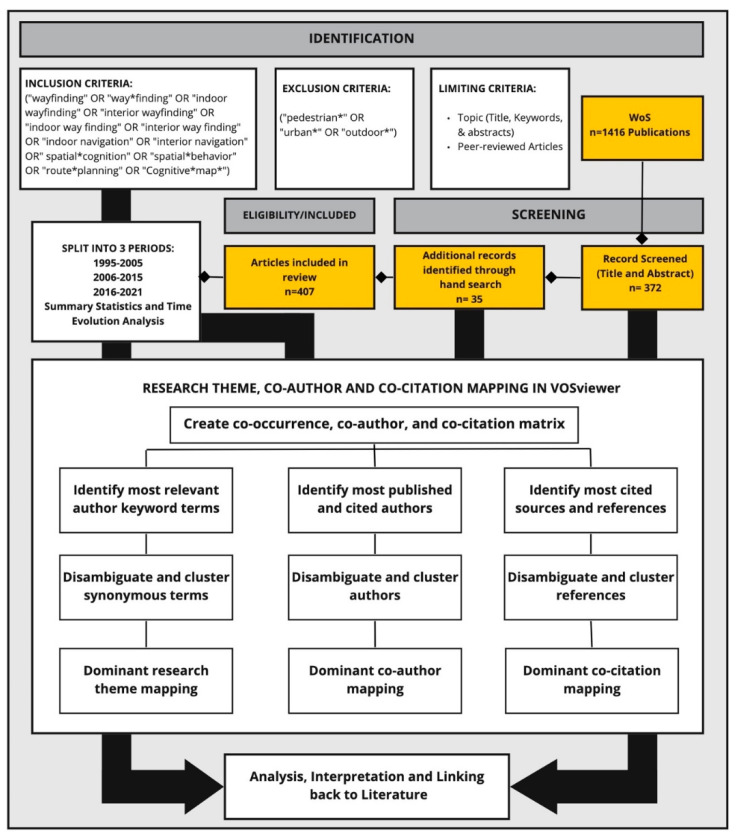
Search strategy and multifaceted bibliometric analysis framework (adapted from [59]).

The final database contained 407 publications (Figure 1). The database was reviewed to identify the temporal patterns in the literature, including dividing it into 3 broad time periods (early research, 1995–2005; developing research, 2006–2015; advanced research, 2016–2021). The time periods were identified based on the distribution of publications to make sure there were sufficient data in each period for further analysis and comparisons.

### 2.2. Analysis Using VOSViewers

Over the past few years, different visualization tools have been used for science mapping and bibliometric analysis [60]. The main objectives of these visualization tools are to demonstrate the dynamic and complex relationships between the fields, authors, journals, organizations, countries, and the core concepts of the knowledge. This study used VOSviewer, a widely-used tool that has a simple and conveniently interpreted graphic interface to create bibliometric networks of authors, publications, journals, organizations, and countries [61]. The networks are generated based on co-occurrence terms, co-authorship, bibliographic coupling, and co-citation analyses (readers who are interested in more details on these methods can refer to [62] for more details). To add more accuracy for the analyses, a thesaurus file was created and adopted in this study (e.g., “cognitive mapping”, “cognitive maps”, and “cognitive-map” all refer to the same terminologies and were combined as “cognitive maps”).

In addition to the co-occurrence analysis, bibliographic coupling analysis of countries and organizations was conducted to detect the leading most organizations and countries that have worked on research in the field. Co-citation analysis was also utilized to identify the most prominent journals and publications and demonstrate their relationships. The output of each of these analyses is a graph network of nodes and links, where larger nodes and thicker links indicate the higher importance of those components. The maps generated by VOSviewer are based on the modularity-based clustering method that includes nodes and links. The node sizes demonstrate the frequency of the considerations, and the thickness of the links displays the strength of the connections between the nodes. Additionally, closely linked nodes form clusters that, for instance, in the case of term co-occurrence analysis, indicate thematic clusters.

## 3. Results

The results of the term co-occurrence analysis, as well as bibliographic coupling, are presented in this section.

### 3.1. Term Co-Occurrence Analysis (Descriptive Data)

**Figure 2:**
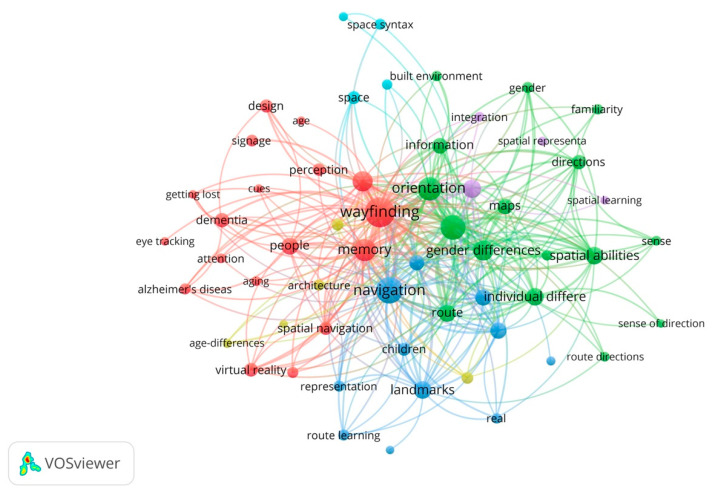
The term co-occurrence map.

To detect the thematic clusters of the topic of indoor wayfinding, term co-occurrence analyses were conducted. This bibliographic method is useful to detect the major core concepts and identified topics, and it can be used to define the thematic clusters [61]. Using the software enabled us to specify the thematic clusters according to the strength of the connections between the terms. The co-occurrence frequency of the terms shapes the clusters. This requires previous knowledge of the field, an understanding of the evolution of wayfinding over time, and the use of advanced technological methods. The output of the VOSviewer analysis is shown in Figure 2. The node sizes are proportional to the number of terms. Some of the general keywords that might have occurred in the publications and were not related directly to the subject were eliminated from the keywords (e.g., “performance”, “strategies”, “model”). The frequency of co-occurrence of the top 20 highly occurring terms is presented in Table 1.

Over the period of 1981–2021, the most common research topics were “wayfinding” (*n* = 152; 14.5%), “navigation” (*n* = 108; 10.3%), “spatial knowledge” (*n* = 93; 8.9%), “orientation” (*n* = 81; 7.8%), and “memory” (*n* = 66; 6.3%) (Table 1). These topics also showed the highest values of total link strength. This shows that these key terms have been paid more attention to by the authors. The terms “wayfinding”, “navigation”, “spatial knowledge acquisition”, and “orientation” showed the higher frequency of co-occurrence, and they appeared near the boundaries of the clusters, which indicates that these are cross-cutting terms that have strong connections to different clusters and terms (Figure 2). Higher values of the terms “cognitive maps” and “memory” imply that indoor wayfinding has been investigated from the cognitive neuroscience perspective. The results also show that the “individual differences”, including gender and age differences, have been largely investigated in the literature relevant to indoor wayfinding.

Figure 2 shows that three major clusters can be identified based on the co-term occurrence analysis. The main two large clusters (red and green) include 34 keywords in total. The largest cluster (red color) comprises 18 keywords and mainly focuses on wayfinding as it relates to “spatial navigation”, “memory”, and “signage”. This cluster shows that there is a close relationship between wayfinding, memory, and signage. Memory has played an essential role in indoor wayfinding literature. Several studies in the fields of neuroscience and cognitive neuroscience suggest that different memory systems are responsible for the two types of spatial memories relevant to wayfinding. The two types of spatial memories are survey knowledge (knowledge of the relationships between landmarks) and route knowledge (which is a form of sequential knowledge acquired based on rewarded responses to stimuli) [63]. Hund [34] found that visuospatial working memory plays an essential role in wayfinding and direction-giving in indoor environments. Bohbot et al. [50] also found that the hippocampal system plays an important role in acquiring survey knowledge (cognitive map) and the caudate nucleus is related to route knowledge. Hölscher et al. [51] found that people who are familiar with an environment and have acquired spatial memories of it can navigate the building using the shortest and fastest route. The impact of signage also has been highlighted in several other studies [1,64,65]. Rousek and Hallbeck [9] found that exposing people to specific color contrasts and symbols can aid or impede recognizing signage for normal or visually impaired people. Some studies indicated that signage placed at decision points increases wayfinding performance [46,66].

This cluster also includes “aging”, “Alzheimer’s disease”, and “dementia” as they relate to navigation errors and spatial disorientation in indoor environments. The difference between this cluster and others is due to the different approaches of disciplines of wayfinding. Previous research shows that aging negatively impacts wayfinding [19,41,44,67]. Rodgers et al. [68] found that aging affects the type of information that people look for, and older adults tend to adopt different navigation strategies than younger adults. Cognitive aging affects wayfinding in the elderly population in terms of orientation and navigation [69]. Moffat et al. [70] indicated that navigational errors are often one of the earliest symptoms of dementia. Marquardt and Schmieg [71] found that people with advanced dementia are more dependent on a compensating indoor environment than those without dementia. Passini et al. [72] concluded that Alzheimer’s disease patients are less able to plan a solution to a complex wayfinding problem, which results in functioning in “incremental and sequential fashion” (p. 687) from one point to another. Interestingly “eye tracking” and “virtual reality” as methods of investigation of wayfinding are included in this cluster.

“Eye-tracking” and “virtual reality” also have been paid attention to in wayfinding in interior environments literature. Ghamari and Pati [73] investigated eye fixations during wayfinding in unfamiliar indoor environments and found that identifying informative signs and architectural features is the most influential visual environmental attribute of wayfinding. Livingstone-Lee et al. [74] conducted an eye-tracking study to objectively investigate the selection of navigational strategies. The results of this study showed there was a clear difference in gaze position in the allocentric (the Place maze) and egocentric (the Cue maze). Kober and Neuper [38] used electroencephalographic (EEG) to investigate sex differences as male and female young adults navigated virtual environments. The results showed a stronger sensorimotor integration in women than in men, possibly due to the differences in cortical theta activity. Lee and Kline [19] used virtual reality to examine wayfinding behavior differences across age groups and found that the older participants tended to acquire environmental information with a higher level of saliency than the younger subjects.

The red cluster of the bibliometric analysis indicates that there is a close relationship between studies that focused on memory as it relates to spatial navigation, Alzheimer’s disease, and dementia due to cognitive decline, particularly in aging populations. Signage, as one of the most influential environmental factors, has been strongly connected with spatial navigation, and virtual reality and eye-tracking methods have been widely used to investigate spatial navigation within indoor environments.

The second-largest cluster (green color) has 16 terms. The main focus of this cluster included “orientation” and “spatial knowledge acquisition” as they relate to “route”, “maps”, and “directions”. This cluster demonstrates the importance of individual differences (mainly gender differences) during wayfinding. Spatial knowledge acquisition first was introduced by Siegel and White [21]. They suggested that the spatial memories include three types of spatial knowledge: (1) landmark knowledge, (2) route knowledge, and (3) survey knowledge. A map is a “diagrammatic, 2-dimensional representation of the global environment” [53] (p. 50). Thorndyke and Hayes-Roth [55] found that individuals who used maps performed better than real-world navigators in estimating the relative locations of and straight-line distances between landmarks. Butler et al. [75] found that participants that used You-Are-Here maps (YAH maps) took a longer time to find a destination. Chen et al. [1] also found that signs were more efficient than YAH maps. There are several studies that investigated the relationship between sense of direction and wayfinding behavioral and navigational performance, and found that participants with a better sense of direction were faster and committed fewer navigational errors [76,77]. The main focus of the green cluster indicates that there is a close relationship between spatial knowledge acquisitors (survey, route, and landmark knowledge) and maps, directions, and routes. The second main focus of this cluster was individual differences, particularly gender differences. Castelli et al. [78] found that males committed fewer navigational errors than females in a pointing task, which suggests that the males acquired greater survey knowledge than the female participants. Choi et al. [35] found that the female participants used landmark knowledge more than the male participants. Studies conducted by Schmitz [36,37] also showed that female participants relied more on landmarks than the male participants in drawing maps and giving directions.

The third-largest cluster (blue color) includes 10 keywords and the main focus of this cluster included “navigation”, “cognitive map”, and “spatial cognition”. It appears that this cluster bridges the other two clusters (red and green). The topic of cognitive mapping has been widely addressed in wayfinding literature [24,46,63]. These topics within this cluster relate to spatial abilities and spatial knowledge acquisition as they occur during navigation (green cluster), and working memory and perception during wayfinding (red cluster). This cluster also consists of studies of indoor wayfinding that involved children. Jansen-Osmann and Fuchs [47] found that children are able to use landmark–location pairings as spatial information like adults when learning an unknown environmental space. Golledge et al. [5] suggested that the development of children’s configurational knowledge structures is not a simple consequence of learning declarative and procedural knowledge systems. Overall, the results of this section show that there is a close relationship between the three clusters identified from co-occurrence analysis.

### 3.2. Core Research Topics and Themes across Different Periods

This section discusses the results of the bibliometric analysis on indoor wayfinding literature and the evolution of the core research topics over time. This literature shows that there is annual growth in publications in this subject area. The subject areas covered by this literature mapping have increased substantially over time [7]. The indoor wayfinding literature can be divided into three periods: early research (1981–2005), developing research (2006–2015), and advanced research (2016–2021). The first recorded publication from this mapping review was published in 1981 and evaluated architectural legibility in the built environment (Weisman, 1981). The findings of the study show that there were only 98 publications (24.1%) over the next 24 years (1981–2005). After 2005, there was fast growth in research on indoor wayfinding, with 130 publications (31.9%) over the following ten years in the developing period (2006–2015), and a much faster increase with a further 179 publications (43.9%) in the advanced period (2016–2021). It should be noted that bibliometric analysis requires a reasonable number of publications from each period. This has also been taken into account when defining the periods.

**Figure 3:**
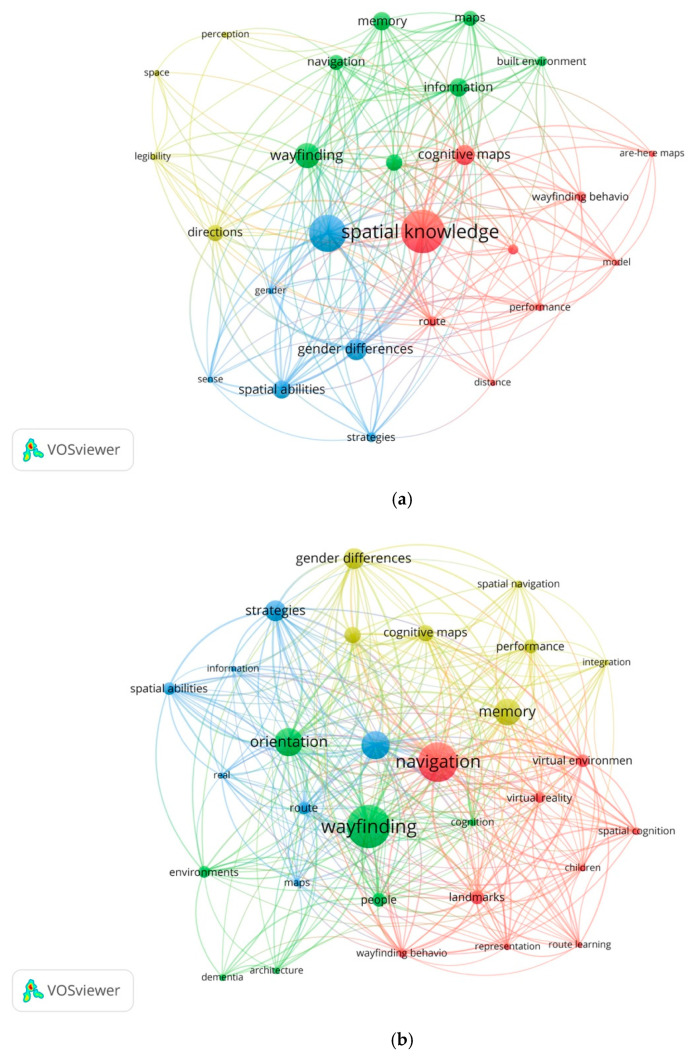

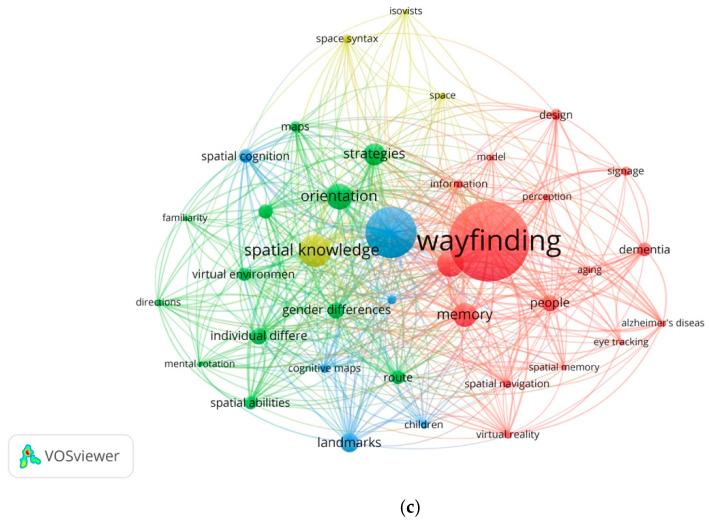
Major themes in the literature based on the term occurrence for each of the three time periods: (**a**) early (1981–2005), (**b**) developing (2006–2015), and (**c**) advanced (2016–2021). The size of the circle is proportional to the occurrence of the keyword, while the line thickness proportional to the strength of co-occurrence.

Drilling down to specific periods shows in detail how some topics have fluctuated over time with upward, downward, and stable trends (Figure 3). Generally, the research has diversified with the increased volume of publications over time. Table 2 shows the 20 most frequently used keywords for early research (1981–2005), developing research (2006–2015), and advanced research (2016–2021). The results show that research concerning “wayfinding”, “orientation”, and “spatial knowledge” have been the primary focus in the literature, with these topics in the top five research priorities through all time periods. The topic of “navigation” also has been one of the primary focus areas since 2006, while from 1991–2005, it ranked 12th. “Navigation” has been used interchangeably with “wayfinding” in the literature and hence, it has received more attention with the growth in the number of publications. Other topics that also have relatively stable positions in terms of research priorities include “memory”, ranking 7th in 1981–2005, 5th throughout 2006–2015, and 6th from 2016 to 2021, “route”, ranking 14th in 1981–2005, 11th (2006–2015), and 12th (2016–2021), and, similarly, “gender differences”, ranking 4th (1981–2005) to 6th (2006–2015) to 8th (2016–2021).

Other topics have progressively increased in terms of research priority through the time periods. “Signage” has received much more attention over time. This topic has gone from 139th (1981–2005) to 41st (2006–2015) to 22nd (2016–2021). It appears that signage, as a visual environmental factor, is emerging as a core topic in indoor wayfinding literature. The results showed that “Individual differences”, went from 26th (1981–2005) to 8th (2006–2015) to 9th (2016–2021). Individual differences are addressed in different studies [36,39,40,44]. The topic has been widely paid attention to in the developing (2006–2015) and advanced research phases (2016–2021). A meta-analysis study conducted by Nazareth et al. [79] provides an overall magnitude of sex differences in large-scale navigation. Similarly, “landmark” has moved from 37th (1981–2005) to 9th (2006–2015) to 7th (2016–2021). From an early period, many studies have paid attention to “landmark”. Jansen-Osmann and Fuchs [47] found that the existence of landmarks influenced the wayfinding performance of adults and children in the same way. Balaban et al. [80] investigated the impact of effect in landmark-based wayfinding. The findings suggested that that the best recognition and wayfinding performance happened when negatively laden landmarks were used. Hamburger and Roser [81] found that nonvisual information may successfully constitute a landmark. Other studies have highlighted the importance of landmarks in spatial navigation [8,82,83,84,85,86].

Research concerning “Alzheimer’s disease” (50th to 45th to 29th) and “dementia” (42nd to 26th to 11th) have increased considerably as priorities over time. In the early research phase (1981–2005), these topics were primarily addressed through the lens of identifying the navigational problems and wayfinding errors through spatial knowledge acquisition [72,87,88,89,90]. In the developing research stage (2006–2015), with more advanced technologies and emerging neuroarchitecture studies, the topics received more attention, primarily focusing on cognition, perception, visual cues, and the role of architectural design [54,71,91,92,93,94]. The research in this field has been widely expanded in the advanced period (2016–2021), with studies investigating the topics in care facilities [42,43,95,96]. Conversely, downtrends are observed for some topics, such as “cognitive maps”, dropping slightly from 5th (1981–2005) to 7th (2006–2015) to 18th (2016–2021), and “map”, slipping from 11th (1981–2005) to 18th (2006–2015) to 17th (2016–2021).

New priority research topics have also been established during the developing (2011–2015) and the advanced period (2016–2021). Signage is a core topic in the advanced period (2016–2021). It appears that studies are focusing more on the impact of visual environmental attributes in the field [53,73,97,98,99,100]. In the developing period, there was a shift towards virtual reality, with novel topics such as “virtual reality” (18th), and “virtual environments” (16th) developing as leading research priorities [17,66,67]. As for the advanced period (2016–2020), new priorities included “eye-tracking” (36th), “isovist” (38th), and “Montreal Cognitive Assessment” (44th). Research using the eye-tracking method has been investigating the visual environmental factors that impact wayfinding behavior [73,74,86,97,101,102,103,104]. Isovists and visibility graph analyses have also been paid more attention recently in indoor wayfinding studies [56,105,106]. The literature also shows that the Montreal Cognitive Assessment has been recently used frequently to measure cognitive abilities during wayfinding [42,102,107].

### 3.3. Prominent Journals

**Figure 4:**
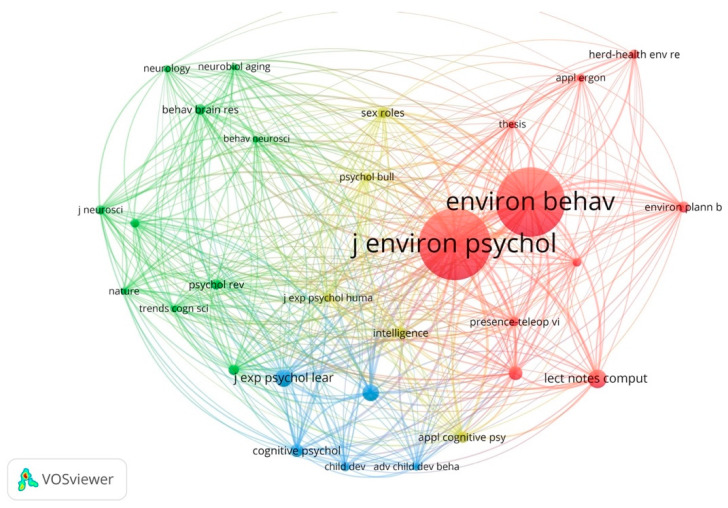
Co-citation analysis by cited sources.

Van Eck and Waltman [108] indicated that “co-citation is a link between two items that are both cited by the same document”. In this section, this analysis was used to identify the journals that have had the highest impact in the field. The details of the total link strengths and citation counts of the most prominent journals can be found in Table S1 of the Supplementary Materials. Figure 4 demonstrates the results of the co-citation analysis by cited sources. The results show that the two main journals in this field are the *Journal of Environmental Psychology* and *Environment and Behavior*. Two major clusters can be identified from the results. The largest cluster (red color) includes journals that are mainly focused on environment and behavior, environmental psychology, and environmental design. The most prominent journals of this cluster are the *Journal of Environmental Psychology*, *Environment and Behavior*, *Health Environments Research and Design Journal*, and *Environment and Planning B*. The second larger cluster of journals (green color) primarily focuses on the relationship between cognitive neuroscience and neurology as they relate to cognitive processes and maps of wayfinding. The most prominent journals of this cluster include *Psychological Review*, *Behavioral Brain Research*, and *Trends in Cognitive Sciences*. The frequency of co-occurrence of the top 20 most cited sources is presented in Table S1.

### 3.4. Prominent Countries

**Figure 5:**
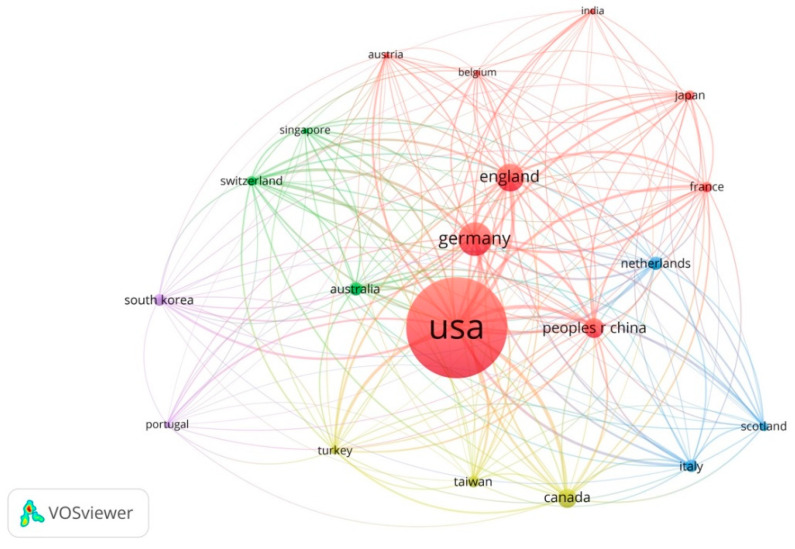
Co-citation analysis by countries.

To identify the most influential countries that have contributed the most to the knowledge in the field, a bibliographic coupling analysis was conducted. “A bibliographic coupling link is a link between two items that both cite the same document” [108] (p. 26). Interested readers can refer to [108] for more details. The results of the bibliographic analysis for the most prominent countries are shown in Figure 5. The sizes of the nodes are proportional to the number of publications. The list of the most prominent countries with the number of documents, number of citations, and total link strength is presented in Table S2 of the Supplementary Material. In this analysis, the countries that had at least five documents relevant to the topics were included. The results of these analyses demonstrate the United States is the most prominent country in the field (with 121 documents, 29.7% of the total documents). The results also show that other developed countries, such as Germany, England, China, and Canada, have had major contributions to the field. The previous studies in the field have shown that cultural differences impact wayfinding ability. For instance, Hashim et al. [65] found that participants from the United Arab Emirates experienced more difficulty understanding healthcare signs than they did understanding general purpose signs. Another study conducted by Lo et al. [109] found similar results that indicated participants in Taiwan had difficulty understanding signage systems that were developed in the United States. Because of the cultural differences, it is recommended that further work from developing countries might be needed to investigate these differences in depth.

### 3.5. Prominent Publications

**Figure 6:**
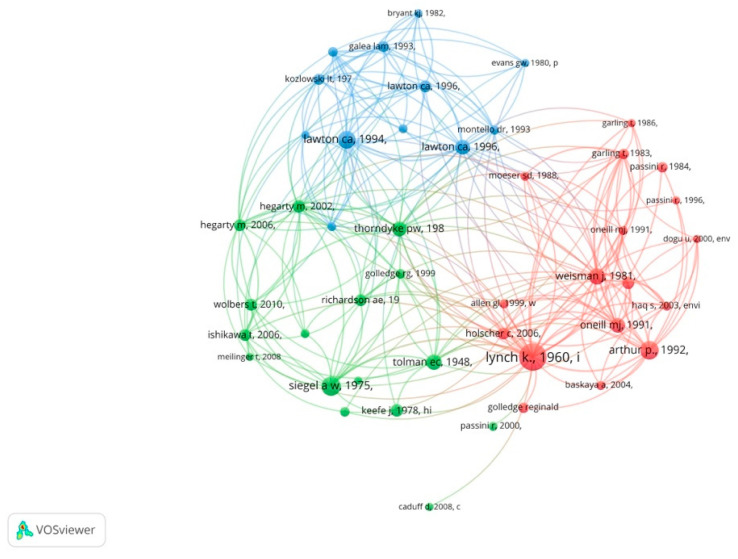
Co-citation analysis by cited references.

Co-citation analysis was also used to identify the most prominent publications in this field. Figure 6 demonstrates the analyses by cited references for a minimum number of 20 citations. The 10 most cited references are shown in Table S3 of the Supplementary Materials. This analysis shows that there are three clusters of the most prominent publications (red, green, and blue). The red cluster includes studies that introduce the theories and principles of wayfinding and spatial knowledge acquisition (e.g., [4,45,49,51]). This cluster associates with the red cluster identified in Section 3.1 (term co-occurrence analysis) as it relates to “spatial navigation”, “memory”, and “signage”. The second-largest cluster (green) consists of some other studies that focus on wayfinding behavior and individual differences between people in navigations (e.g., [21,25,55,110,111,112]). This cluster relates to the green cluster identified in Section 3.1 (term co-occurrence analysis) and primarily focuses on individual differences during indoor wayfinding. The cluster in blue includes two major types of documents that have played foundational roles in investigating wayfinding. First, the documents focus on personality and sex differences in wayfinding behavior (e.g., [39,113,114]). There is a close relationship between this type of document and the green cluster identified in Section 3.1. The second type of document focuses on strategies of wayfinding and spatial knowledge ((e.g., [40,48]). This second type of document of this cluster also associates with the blue cluster found in Section 3.1.

### 3.6. Prominent Authors

**Figure 7:**
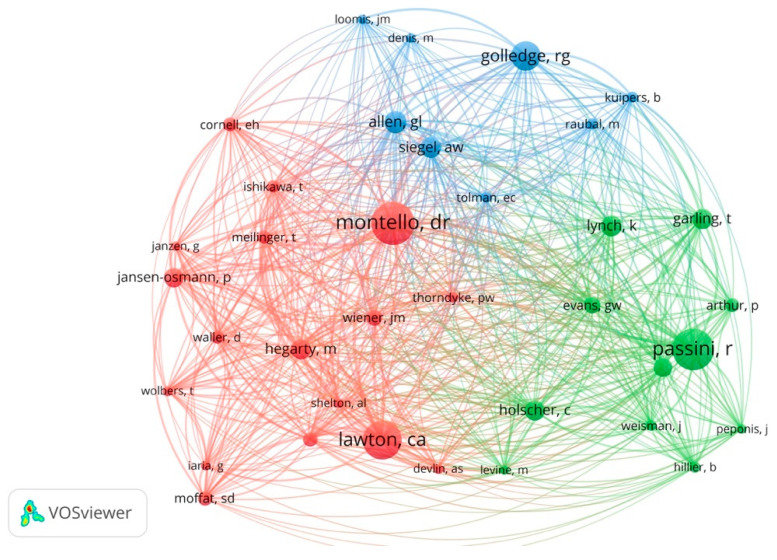
Co-citation analysis by authors.

The co-citation analysis using cited authors was conducted to identify the most influential authors in indoor wayfinding literature. The list of the 30 most prominent authors with a minimum of 20 citations is presented in Table 3. Figure 7 also demonstrates the most influential authors related to indoor wayfinding. Three main clusters can be identified according to the author’s expertise. The results of this analysis are, to a great extent, consistent with the results of the previous section analysis (Prominent Publications). The green cluster includes authors that have mainly worked on the nature of wayfinding and the syntax of space and its impact on navigation (such as Romedi Passini, Kevin Lynch, Paul Arthur, Christoph Hölscher, Tommy Garling, Jerry Weisman, John Peponis, and Bill Hillier). The red cluster of this analysis includes the authors that have expertise in investigating individual differences (gender and sex) and children’s wayfinding behavior (e.g., Daniel Montello, Petra Jansen-Osmann, Carol Lawton, Mary Hegarty, Scott Moffat). This cluster also includes some studies by authors that focus on differences in spatial knowledge, route learning, and the role of landmarks, such as Perry Thorndyke, Jan Wiender, Toru Ishikawa, and Tobias Mellinger. The third-largest cluster (blue) includes authors whose expertise revolve around spatial knowledge, place recognition, and cognitive maps, such as Reginald Golledge, Alexander Siegel, Gary Allen, and Martin Raubal.

## 4. Discussion

This paper has investigated the evolution of and current trends in peer-reviewed indoor wayfinding. By using a bibliometric method, we have captured geographical representation and temporal trends in research priority topics and provided a review of the most cited papers, authors, foundational journals, and research collaboration clusters. Overall, the subject of wayfinding in indoor environments has been given attention across different journals, sectors, and disciplines, with a rapid annual growth rate. The key topics have clearly diversified over time, with new topics such as signage, virtual environments, isovists, and eye-tracking, in particular, in the last ten years.

### 4.1. Theoretical Implications

Even though the topic of wayfinding has drawn attention in the literature, there is a lack of knowledge in investigating the evolution and trends of indoor wayfinding over time. This review is meant to develop the field’s understanding of indoor wayfinding. This paper has provided a broad assessment of the overall trends in peer-reviewed indoor wayfinding science. In particular, this assessment allows for tracking progress toward achieving a better understanding of indoor wayfinding and may provide designers, architects, and practitioners with useful insights into the trends and trajectories in the field. The main objective of this study was to provide an overview of the literature on indoor wayfinding assessment. The findings of the study show that a large body of literature has been published over the last decade, with a rapid increase in the number of publications since 2016. Our analysis has looked at the geographical distribution of knowledge contribution, co-authorship linkages, tracked changes in research priority themes over time, and examined the most influential journals, papers, and authors that underlie the literature. The knowledge mapping and analysis of the evolution of indoor wayfinding provided in this paper is an attempt to create a universal language and to provide a framework for managing and organizing the literature, as well as to provide suggestions for future studies. This review intends to deepen the understanding of wayfinding and its factors that can serve as a resource for designers, healthcare administrators, and practitioners.

The first recorded publication from this mapping review was published in 1981 and evaluated the architectural legibility in the built environment [49]. The findings of this study show that there were only 98 publications (24.1%) over the next 24 years (1981–2005). After 2005, there was fast growth in research on indoor wayfinding, with 130 publications (31.9%) over the following ten years in the developing period (2006–2015), and a much faster increase with a further 179 publications (43.9%) in the advanced state of the most recent five years (2016–2021). This shows that there is an average annual growth in publications in this subject area. The subject areas covered by this literature mapping have increased substantially over time. The results of this study show that while there is a rapid increase in the number of publications over time, the wayfinding in indoor environment literature is dominated by nine countries (the United States, Germany, England, China, Canada, Australia, the Netherlands, Italy, and South Korea), which together comprise 67.1% of all publications, raising questions about the representativeness of this knowledge. This dominance is not surprising, given the capacities and early and long-term investments in the research of wayfinding in indoor environments across these countries. The cultural differences and their impact on perceiving the environmental factors such as signage, maps, and directories, as well as spatial layout, suggest more research in developing countries and different settings needs to be conducted.

Despite the rapid growth in recent years, the start of indoor wayfinding literature was slow. As our results demonstrate, the early phase saw a limited number of publications. Before the 1990s there were only a handful of publications specifically focusing on indoor navigation wayfinding (e.g., [21,49,55,115,116]). Several fundamental papers in the 1990s (e.g., [18,46,48,75,88,117,118,119]) started bringing more focus to indoor wayfinding. These studies primarily focused on spatial knowledge acquisitions through maps, route knowledge, survey knowledge, and landmarks. Additionally, some other studies (e.g., [39,40,41,44,113,114]) focused on the individual differences (personality, age, and gender) in wayfinding behavior. The results in gender differences in wayfinding behavioral performance were inconsistent. Some studies found there is no difference between males and females in wayfinding, and some studies reported that males are able to perform better.

### 4.2. Practical Implications

The results of this study can shed light on the evolution of indoor wayfinding literature. The findings help to better understand the trends, tools, approaches, and methods of indoor wayfinding research. A better understanding of the wayfinding phenomenon can allow designers to make better and more effective predictions about how design decisions impact the health and well-being of the users of healthcare facilities. This review can inform the researchers of the prominent key concepts, journals, authors, and organizations, and therefore, can serve as a point of reference for a deeper understanding of the field. The advanced methods of virtual and augmented reality, eye-tracking, and recognitions using artificial intelligence allow the researchers to further investigate the complex behavior of wayfinding in different settings with various groups of participants. Additionally, the results indicate the visual environmental attributes, such as signage, have received more attention in recent years, and future studies can investigate this topic further. By examining three periods (1981–2005, 2006–2015, and 2016–2021), it was found that in the first period, the intellectual foundations of the knowledge of indoor wayfinding were diverse but fragmented. In the last two periods (2006–2015 and 2016–2021), there is some indication of consolidation. Wayfinding is a multifaceted discipline with a dynamic nature, and because of this, the diversity of the intellectual foundation is not surprising.

### 4.3. Limitations

Despite the fact that bibliometric analysis is an innovative method for analyzing large quantities of data, the results must be interpreted with caution because of the different limitations of the study. First, while VOSviewer is shown to be an efficient tool for visualization and literature mapping, particularly with the rising numbers of publications, this does not indicate that bibliometric analysis and mapping can replace integrative and systematic reviews. While this study was conducted primarily on publications titles, abstracts, and keywords for the screening process, advanced techniques in literature mining and machine learning in the future and analyses of the full-text reviews of the publications, might improve in-depth analysis along with systematic reviews. It should be noted that the interpretations of the term co-occurrences and thematic clusters should be viewed with caution due to the fact that the frequency of the term co-occurrences and the connections between the terms might not be sufficient to specify the relationships between the terms. The term co-occurrence cluster analysis of the current study is only based on the number of times that the terms co-occurred and does not necessarily provide any explanations on the nature of the relationships between the terms. However, the themes and trends identified from the bibliometric mapping review guide to conduct more in-depth and systematic reviews. Bibliometric analysis studies are different from systematic reviews, as they serve to demonstrate the overall landscape of the research and mapping the knowledge. It should be noted that some of the sub-topics highlighted in this study require more in-depth analysis through a systematic review. This study does not replace systematic reviews in this field of study, but it complements them. The other limitation of the study is that the bibliometric reviews primarily pay attention to the quantitative numbers of the publications, citations, and term occurrences, and more in-depth detailed reviews are required to address the publication quality issues. Another limitation for this study that must be noted is that while the aim of this study is to provide an overview of the research landscape, a more in-depth analysis should focus on healthy adult populations, as the topic of wayfinding is a complex concept for various populations, including children, and dementia and Alzheimer’s disease patients.

### 4.4. Conclusions

While this current bibliometric review has provided insights into advancing our comprehension of the literature on wayfinding in indoor environments and identifying the evolution patterns, future studies are recommended to better comprehend more specific issues. Future studies may also investigate how emerging trends and core concepts can be better integrated into wayfinding and navigation assessment. Finally, this study only included peer-reviewed articles. The future research could include books, book chapters, reports, conference proceedings papers, and other publications. Currently, the bibliometric methods only analyze documents that are indexed in scientific databases, such as Scopus or the Web of Science. Future research could also look into similar trends in grey literature to capture the implementation of experiences across the world, provide additional insights into how indoor wayfinding has evolved as a topic over time, and identify future directions on emerging trends.

## Figures and Tables

**Table 1:** Term co-occurrence analysis (top 20 most frequently occurring terms).

Keyword	Occurrences	Percentage	Total Link Strength
Wayfinding	152	14.5	1362
Navigation	108	10.3	1069
Spatial Knowledge	93	8.9	952
Orientation	81	7.8	817
Memory	66	6.3	777
Environments	53	5.1	576
Gender Differences	53	5.1	605
Cognitive Maps	40	3.8	402
Individual Differences	39	3.7	451
Spatial Abilities	39	3.7	429
Landmarks	38	3.6	424
Route	35	3.3	416
People	34	3.3	405
Spatial Cognition	30	2.9	325
Virtual Environments	30	2.9	358
Maps	29	2.8	270
Information	27	2.6	251
Wayfinding Behavior	26	2.5	286
Dementia	23	2.2	232
Directions	23	2.2	236

**Table 2:** The 20 most frequently used keywords for early research (1981–2005), developing research (2006–2015), and advanced research (2016–2021).

	1981–2005			2006–2015			2016–2021		
	Keywords	*n*	%	Keywords	*n*	Percentage	Keywords	*n*	%
1	Spatial Knowledge	26	7.7	Wayfinding	44	9.2	Wayfinding	93	11.1
2	Orientation	22	6.5	Navigation	40	8.4	Navigation	59	7.0
3	Wayfinding	15	4.4	Orientation	29	6.1	Spatial Knowledge	38	4.5
4	Gender Differences	13	3.8	Spatial Knowledge	29	6.1	Environments	31	3.7
5	Cognitive Maps	12	3.5	Memory	27	5.7	Orientation	30	3.6
6	Information	11	3.2	Gender Differences	21	4.4	Memory	28	3.3
7	Memory	11	3.2	Cognitive Maps	17	4.4	Landmark	22	2.6
8	Spatial Abilities	11	3.2	Individual Differences	17	3.6	Gender Differences	19	2.3
9	Directions	10	2.9	Landmark	14	3.6	Individual Differences	19	2.3
10	Environments	10	2.9	People	14	2.9	People	19	2.3
11	Maps	9	2.7	Route	13	2.9	Dementia	16	1.9
12	Navigation	9	2.7	Spatial Abilities	13	2.9	Route	16	1.9
13	Built Environment	6	1.8	Virtual Environments	13	2.7	Spatial Cognition	16	1.9
14	Route	6	1.8	Environments	12	2.7	Spatial Abilities	15	1.8
15	Spatial Cognition	6	1.8	Virtual Reality	11	2.7	Virtual Environments	15	1.8
16	Wayfinding Behavior	6	1.8	Wayfinding Behavior	9	2.5	Design	13	1.5
17	You-Are-Here Maps (YAHM)	4	1.2	Cognition	8	2.3	Maps	12	1.4
18	Distance	4	1.2	Maps	8	1.9	Cognitive Maps	11	1.3
19	Gender	4	1.2	Spatial Cognition	8	1.7	Signage	11	1.3
20	Legibility	4	1.2	Spatial Navigation	8	1.7	Space Syntax	11	1.3

**Table 3:** Highly cited authors.

Author	Citations	Total Link Strength
Montello, DR	634	57
Höelscher, CH	493	83
Golledge, RG	479	23
Lawton, CA	473	55
Oneill, MJ	299	55
Wiener, J	248	34
Ishikawa, T	189	16
Passini, R	170	17
Rainville, C	170	17
Marchand, N	162	16
Raubal, M	148	9
Hund, A	146	34
Noriega, P	144	49
Rebelo, F	144	49
Vilar, E	144	49
Blades, M	99	66
Courbois, Y	99	66
Farran, E	99	66
Janzen, G	98	12
Jansen-Osmann, P	73	29
Raubal, M	47	399

## Data Availability

Not applicable.

## Supplementary Materials

The following are available online at https://www.mdpi.com/article/10.3390/ejihpe11020042/s1: Table S1: Co-citation analysis by cited sources; Table S2: Countries with the largest number of publications; Table S3: Ten most cited references.
