# Supplementary material for: Mapping the Evolutions and Trends of Literature on Wayfinding in Indoor Environments
Source: Eur J Investig Health Psychol Educ. 2021 Jun 18;11(2):585–606. doi: 10.3390/ejihpe11020042 (PMC8314368; doi:10.3390/ejihpe11020042)
Supplement: Supplementary file 1 [file ejihpe-11-00042-s001.zip › ejihpe-1246114-supplementary.pdf]

Supplementary material for the article titled:

Mapping the Evolutions and Trends of Literature on Wayfinding in Indoor Environments

Items in this Supplementary document:

- The search string
- Table S1: Co-citation analysis by cited sources (top 20 most influential journals)
- Table S2: Countries with the largest number of publications.
- Table S3: Ten most cited references

The search string:

TS= (("wayfinding" OR "way*finding" OR "indoor wayfinding" OR "interior wayfinding" OR "indoor way finding" OR "interior way finding" OR "indoor navigation" OR "interior navigation" OR" spatial*cognition" OR "spatial*behavior" OR "route*planning" OR "Cognitive*map*") NOT ("pedestrian*" OR "urban*" OR "outdoor*"))

Table S1. Co-citation analysis by cited sources (top 20 most influential journals)

Source	Citations	Total link strength
Journal of Environmental Psychology	679	11805
Environment and Behavior	658	9495
Lecture Notes in Computer Science	179	2008
Journal of Experimental Psychology: Learning, Memory, and Cognition	164	3448
Memory and Cognition	161	3564
Spatial Cognition and Computation	132	2825
Cognitive Psychology	125	2442
Applied Cognitive Psychology	108	2223
Environment Planning B	108	1520
Psychological Review	104	1657
Cognition	103	2095
Intelligence	101	2047
Sex Roles	101	2351
Behavioral Brain Research	100	2085
Health Environments Research and Design	92	652
Presence Teleoperators and Virtual Environments	92	1389
Child Development	89	1869
Journal of Neuroscience	89	1855
Image city	87	1246
Advances in Child Development and Behavior	80	1247

Table S2. Countries with the largest number of publications.

Country	Documents	Citations	Total link strength
Usa	121	3501	24650
Germany	41	986	13559
England	34	522	8430
China	24	176	6794
Canada	23	537	5867
Australia	16	246	2894
Netherlands	16	165	3693
Italy	15	130	3250
South Korea	14	78	1781
Taiwan	14	162	2067
France	13	147	4100
Japan	12	359	2422
Switzerland	12	296	5942
Scotland	11	608	2368
Turkey	10	309	4371
Austria	7	246	1477
Portugal	7	128	1020
Singapore	7	41	1861
Belgium	6	27	1213
India	5	13	701

Table S3. Ten most influential publications.

Title	Co-citations	total link strength	Reference
The Image of the City	79	441	[45]
The Development of Spatial Representations of Large-Scale Environments	54	275	[21]
Wayfinding: People, Signs, and Architecture	51	281	[4]
Gender differences in way-finding strategies: Relationship to spatial ability and spatial anxiety	49	398	[39]
Evaluating Architectural Legibility: Way-Finding in the Built Environment	44	329	[49]
Cognitive Maps in Rats and Men	41	257	[25]
Strategies for Indoor Wayfinding: The Role of Orientation	40	350	[40]
Differences in spatial knowledge acquired from maps and navigation	40	310	[55]
Effects of Signage and Floor Plan Configuration on Wayfinding Accuracy	39	282	[46]
Development of a self-report measure of environmental spatial ability	33	295	[113]
